# Changing Wheat Bran Structural Properties by Extrusion-Cooking on a Pilot and Industrial Scale: A Comparative Study

**DOI:** 10.3390/foods10020472

**Published:** 2021-02-21

**Authors:** Chiara Roye, Muriel Henrion, Hélène Chanvrier, Chrystel Loret, Roberto King, Lisa Lamothe, Christophe M. Courtin

**Affiliations:** 1 Laboratory of Food Chemistry and Biochemistry and Leuven Food Science and Nutrition, Research Centre (LFoRCe), KU Leuven, Kasteelpark Arenberg 20, 3001 Leuven, Belgium; chiara.roye@kuleuven.be; 2 Société des Produits Nestlé S.A., Nestlé Research and Development Orbe, Route de Chavornay 3, 1350 Orbe, Switzerland; Muriel.henrion@rdor.nestle.com (M.H.); Helene.chanvrier@rdor.nestle.com (H.C.); chrystel.loret@rd.nestle.com (C.L.); 3 Société des Produits Nestlé S.A., Nestlé Research, Vers-chez-les-Blanc, 1026 Lausanne, Switzerland; rokisol@hotmail.com (R.K.); lisa.lamothe@rdls.nestle.com (L.L.)

**Keywords:** extrusion-cooking, bran-water interaction, dietary fibre, wheat bran, extruder-scale

## Abstract

Extrusion-cooking can be used to change the techno–functional and nutrition-related properties of wheat bran. In this study, pilot-scale (BC21) and industrial-scale (BC45) twin-screw extrusion-cooking using different types of extrusion (single-pass, double-pass and acid extrusion-cooking) and process parameters (temperature, moisture) were compared for their impact on wheat bran. When applying the same process settings, the higher strong water-binding capacity, extract viscosity and extractability displayed by bran extruded using the industrial set-up reflected a more considerable wheat bran structure degradation compared to pilot-scale extrusion-cooking. This was attributed to the overall higher specific mechanical energy (SME), pressure and product temperature that were reached inside the industrial extruder. When changing the type of extrusion-cooking from single-pass to double-pass and acid extrusion-cooking, wheat bran physicochemical characteristics evolved in the same direction, irrespective of extruder scale. The differences in bran characteristics were, however, smaller on industrial-scale. Results show that the differentiating power of the latter can be increased by decreasing the moisture content and increasing product temperature, beyond what is possible in the pilot-scale extruder. This was confirmed by using a BC72 industrial-scale extruder at low moisture content. In conclusion, the extruder scale mainly determines the SME that can be reached and determines the potential to modify wheat bran.

## 1. Introduction

Wheat (*Triticum aestivum* L.) bran is a byproduct of the grain roller-milling process and is mostly used as an animal feed ingredient. It consists of botanical bran, aleurone and some residual endosperm. Wheat bran is a concentrated source of dietary fibre, mostly arabinoxylan (AX), as well as vitamins, minerals and other bioactive compounds. The consumption of wheat bran is therefore linked to different positive health effects. First, the physical presence of the bran in the colon and the water-binding by the cell wall matrices and the capillary structures in wheat bran [1,2] can lead to an increased faecal bulk and normalised transit time [3]. Fermentation of dietary fibre by colon bacteria is another major mechanism by which bran mediates health [4]. The produced short-chain fatty acids (SCFA) can result in improved gut health and can also influence metabolic health [5]. SCFA are moreover speculated to have a mediational role in the microbiota–gut–brain axis crosstalk [6]. In wheat bran, nevertheless, only a small portion of the dietary fibre fraction is soluble and readily fermentable [7,8]. Other important constituents of wheat bran are minerals, which are key for the proper functioning of the human body [9]. Due to the presence of phytate, only 5% to 10% of the iron and zinc in wholemeal flour is estimated to be accessible [10]. Moreover, these and other nutritionally relevant compounds are enclosed in the aleurone behind thick, rigid cell walls, making them only accessible to a limited extent for absorption in the small intestine [11]. In such a context, it is desirable to apply modification techniques that increase the interaction of bran with water, open aleurone cells, degrade phytate and increase the bran soluble dietary fibre content. Potential techniques include particle size reduction, bioprocessing and extrusion-cooking [12,13,14]. 

It was previously shown that extrusion-cooking, a food processing strategy combining high shear, pressure and temperature, is a promising technique for the modification of wheat bran properties. It results in the solubilisation of AX, the opening of aleurone cells, degradation of phytate and release of ferulic acid. In previous studies, the extent of the effects was shown to depend on the applied process parameters such as moisture contents, set temperature, screw speed and configuration design [14,15,16,17,18,19]. However, the extrusion-cooking experiments described in these studies were performed on different extruder sizes going from a lab-scale extruder with a feed rate of 1.3 to 3 kg/h [16,17] to a BC45 extruder with a feed rate of 20–32 kg/h [15], making comparison difficult. Moreover, most development work is initially done on small-scale extruders, and the focus during upscaling of extrusion-cooking is mostly on keeping product properties constant. Three strategies exist to achieve this, i.e. volumetric scale-up, power scale-up and heat transfer scale-up. When these strategies are used, residence time, specific mechanical energy (SME) or heat transfer are kept constant [20]. However, if one’s aim is to degrade the wheat bran structure as much as possible, it seems relevant to seek extreme settings in every extruder. Furthermore, it can be questioned if the influence of process settings and type of extrusion-cooking on wheat bran characteristics can be generalised for all extruder types or sizes. 

Against this background, the first aim of this study was to evaluate the effect of upscaling using the same extrusion settings but different extruder sizes on wheat bran properties. Wheat bran was extruded using a pilot-scale extruder (centreline distance of 21 mm) and an industrial-scale extruder (centreline distance of 45 mm) using a comparable screw design and keeping the length over diameter ratio (L/D) constant. Different extrusion types (single-pass extrusion-cooking, double-pass extrusion-cooking and acid extrusion-cooking) were tested. These types of extrusion-cooking were chosen as they were proven to bring interesting modifications to wheat bran functionality [14,21]. Wheat bran characteristics compared after extrusion-cooking included bran extractability, extract viscosity and strong water-binding capacity (SWBC). These parameters best represent the extent of structure degradation of wheat bran and are most relevant from a techno–functional and physiological point of view. SWBC gives an indication of the extent of structure opening occurring during "flash-off" at the extruder outlet. When the product exits the extruder, a pressure drop to atmospheric pressure at high product temperature leads to an instant and explosive water evaporation. This typically leads to the expansion of the product [22]. The water-binding capacity of wheat bran was shown to be important for faecal bulking, which has a positive effect on health [1]. It can furthermore be considered as a rough estimator for the accessibility of the wheat bran structure for gut microbiota, which is of importance for fermentation in the colon [8,23]. Extract viscosity and extractability were measured as an indication for structure breakdown and solubilisation of molecules.

The second aim of this study was to determine the maximal potential of a pilot-scale extruder and an industrial-scale extruder to modify wheat bran. Extrusion at both scales was compared with regard to the system parameters that were achieved (SME, pressure, product temperature) and the potential to change the physicochemical properties of wheat bran (extractability, extract viscosity, SWBC). Extrusion-cooking on a large industrial extruder (centreline distance of 72 mm) was performed as a case study to validate the conclusions about upscaling from pilot to industrial scale. 

## 2. Materials and Methods

### 2.1. Materials

Native wheat (*Triticum aestivum* L.) bran was purchased from Dossche Mills (Deinze, Belgium). It contained 11.8% starch, 20.9% proteins, 5.4% lipids, 24.4% water-unextractable AX, 0.6% water-extractable AX, 9.1% cellulose, 2.1% (1,3:1,4)-β-glucan, 3.3% fructan, 4.3% phytate and 6.4% ash on a dry matter (dm) basis [8]. The average particle size of the wheat bran was 1420 µm. Chemicals, solvents and reagents were purchased from Sigma-Aldrich (Bornem, Belgium). 

### 2.2. Pilot-Scale Extrusion-Cooking with the BC21 Extruder

**Figure 1:**
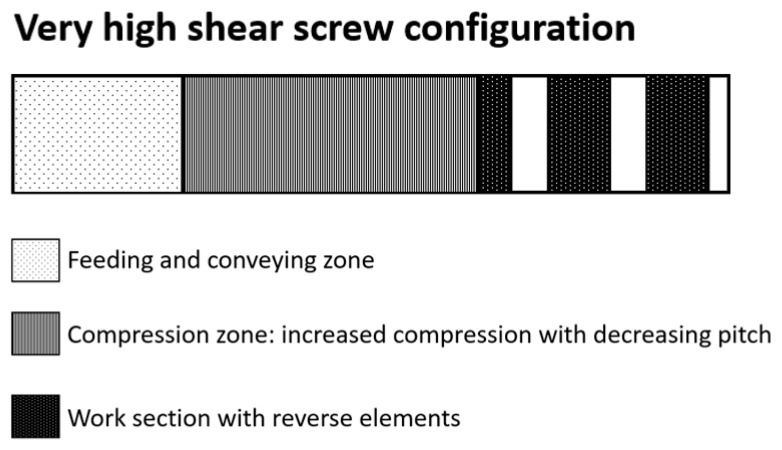
Graphical representation of the very high shear screw configuration used for extrusion-cooking on pilot-scale and industrial-scale.

Wheat bran was extruded in a BC21 twin-screw extruder (Clextral, Firimini, France), with a screw diameter of 25 mm (centreline distance: 21 mm). The extruder consisted of 5 barrels each of 100 mm in length. The length over diameter (L/D) ratio of the extruder was hence 20. A circular die of 3 mm was used. The very high shear screw configuration (Figure 1), as described by Roye, Henrion et al. [14], was used. The feed rate of wheat bran was set at 7 kg/h and the screw speed at 310 rpm. Different levels of water were added directly to the extruder at the beginning of the first barrel, resulting in total moisture contents of 23% and 27%, with 23% being the lowest moisture content that could be reached in the BC21 while ensuring a stable production process. Two different temperature profiles were used: a low-temperature profile (20, 80, 105, 115 and 120 °C in the different barrels) to reach a last barrel set temperature of 120 °C, and a high-temperature profile (20, 90, 120, 135 and 145 °C) to reach a last barrel set temperature of 145 °C. The latter was the highest set temperature that could be used in the last barrel during BC21 extrusion-cooking while ensuring a stable production process. Moisture content and barrel temperature are further referred to as process parameters. For further reference, the samples produced in the BC21 are coded as detailed in Table 1. Roye, Henrion, et al. [14] previously discussed sample physicochemical characteristics, which are summarised in Table S1. Single-pass twin-screw acid extrusion-cooking was performed with 2% citric acid in the final product (VHSA2%) using processing conditions described in Table 1 and by Roye, Chanvrier et al. [21]. For the double-pass extruded samples, the first extrusion pass was performed at 27% of moisture with the very high shear screw configuration and using 145 °C as the fixed last barrel temperature. This was followed by a milling step (Frewitt, Switzerland) to reach an average product particle size of 700 µm before performing the second extrusion step using the same process parameters, yielding sample VHSD27 (see Table 1). Sample physical characteristics and chemical composition are described by Roye, Chanvrier, et al. [21] and given in Table S1. Extrusion-cooking experiments were conducted once, with a run time of at least 30 min after stabilisation. The system parameters, i.e., pressure at the die, specific mechanical energy (SME) and product temperature were measured for all samples. The SME was determined by measuring the torque (Nm) at a constant screw speed using the following formula:

SME = [2*π*Torque*Screw speed]/flowrate of the product

The die pressure was measured in the last barrel of the extruder and is referred to as outlet pressure. The product temperature is the temperature measured in the last barrel. The samples were dried in a convection oven (Wiesheu, Großbottwar, Germany) for 20 min at 120 °C and milled with the Cyclotec 1093 Sample Mill (FOSS, Högenäs, Sweden) using a 0.8-mm sieve to obtain samples with a D50 between 350 and 400 µm. The moisture content of the extruded samples was determined using AACC (American Association of Cereal Chemists) International method 44–15.02 [24]. 

Native bran was included in the experiments as a control sample. It was dried in the convection oven (20 min, 120 °C) and milled with the Cyclotec 1093 Sample Mill (FOSS, Högenäs, Sweden) using a 0.5-mm sieve to obtain samples with a D50 comparable to the extruded samples.

### 2.3. Industrial-Scale Extrusion-Cooking with the BC45 Extruder

Wheat bran was also extruded in a BC45 twin-screw extruder (Clextral, Firimini, France). This extruder has a barrel diameter of 53 mm (centreline distance 45 mm) and 6 barrels, each 191 mm and equipped with individual temperature control. The L/D ratio of the extruder was 21.6. The extruder had a gap of 2 mm, a pre-expansion die of 7 mm and a die of 3 mm. The wheat bran feed rate to achieve equivalence with the BC21 extruder was approximated according to a volume scale-up rule: Q2/Q1 = (D2/D1)^3^, with Q the feed rate and D the screw diameter [25]. Subscript 1 refers to the BC21 extruder and 2 to the BC45 extruder. The calculated feed rate was 67 kg/h. Due to possible variations in the feed rate, the feed rate was set at 70 kg/h. The screw speed was set at 310 rpm. A low-temperature profile was applied in the extruder (20, 80, 105, 115, 115 and 120 °C in the different barrels), reaching a last barrel set temperature of 120 °C. The screw configuration design was upscaled from the BC21 and similarly contained work sections with reverse elements to increase shear as much as possible (Figure 1). 

Based on the results of Roye, Henrion et al. [14] and Roye, Chanvrier et al. [21] on the BC21 extruder, four extrusion-cooking conditions were selected for use with the BC45 extruder and led to the production of samples VHS23, VHS27, VHSD27 and VHSA2% (Table 1). VHS23, VHSA2% and VHSD27 were produced as their BC21 counterparts had the highest extractable dietary fibre content [21], showing structure degradation due to extrusion-cooking. VHS27 was produced as an extra control sample for VHSA2%, both being extruded at 27% total moisture. In addition, this sample allowed evaluation of the effect of moisture content during single-pass extrusion-cooking on an industrial scale when compared with VHS23. For double-pass extrusion-cooking, a first extrusion pass was performed at 23% moisture (VHS23), and a second pass at 27% moisture. 

Aiming for a maximal structure degradation of wheat bran, two additional extrusion-cooking trials were performed with (i) the lowest moisture content that could be reached using 120 °C as last barrel set temperature, i.e., 19% (VHS19, see Table 1) and (ii) the highest set temperature that could be reached in the last barrel, i.e., 145 °C. In the latter case, wheat bran was first preconditioned to 20% of moisture and afterwards extruded at a total moisture content of 27% using the temperature profile of 20–100–110–125–135–145 °C (VHS27T, see Table 1). 

Extrusion-cooking experiments were conducted once, with a run time of at least 30 min after stabilisation. During extrusion-cooking, system parameters were measured as described in Section 2.2. After extrusion-cooking, samples were dried using a fluidised bed dryer until their moisture content was lower than 3%. Samples were cooled afterwards and milled as described above. 

### 2.4. Industrial-Scale Extrusion-Cooking with the BC72 Extruder

Extrusion-cooking of wheat bran on the BC72 industrial-scale extruder (Clextral, Firimini, France) was equally performed. The BC72 extruder has a barrel diameter of 88 mm (centreline distance of 72 mm) and 7 barrels. The L/D ratio of the extruder was 23.9. Every barrel was equipped with an individual temperature control system. The temperature profile used was 30, 80, 100, 100, 100, 100, 90 °C, which was the highest set temperature profile that could be reached. The extruder had a pre-expansion die of 14 mm and a die of 5 mm. The feed rate of wheat bran, estimated according to the volume scale-up rule, as explained in Section 2.3, was set at 300 kg/h. A screw speed of 200 rpm was used. The screw configuration was upscaled from the very high shear design used in the BC21 (Figure 1). To determine the maximal potential of the BC72 extruder to modify wheat bran, the sample with the lowest total moisture content that could be reached during the extrusion-cooking process, i.e., 21.5% of moisture, was produced (Table 1). The extrusion-cooking experiment was performed once, with a run time of 30 min. System parameters were measured as detailed in Section 2.2. The sample was dried, cooled and milled afterwards as described above (Section 2.3). 

It has to be remarked that extrusion-cooking experiments were conducted once as the study is an exploratory study and priority was given to producing samples using different process parameters and different extrusion-cooking types. 

### 2.5. Determination of Bran Extractability

The procedure to determine the extractability of bran is based on AACC International method 56.11.02 [26] and is described by Roye, Bulckaen et al. [8]. An amount of 30.0 mL of water was added to 1.0 g of bran. This suspension was shaken (150 rpm, 30 min, room temperature) and centrifuged (4000× *g*, 10 min, room temperature), followed by the removal of the supernatant. The residue was dried overnight in an oven at 90 °C and the residue was weighed after cooling. The analysis was performed in triplicate for every sample. 

### 2.6. Determination of Bran Extract Viscosity

A bran extract was made by adding 30.0 mL of water to 4.0 g of bran (in triplicate). The suspension was shaken (150 rpm, 30 min, room temperature), centrifuged (4000× *g*, 10 min, room temperature) and the supernatant was separated from the pellet. The supernatant was brought into the sample holder of the HR-2 Discovery Hybrid Rheometer with a Standard Peltier Concentric cylinder and a DIN Rotor (TA instruments). Shear was measured at shear rates of 10 1/s to 100 1/s and 20 °C. As the extract displayed Newtonian behaviour, viscosity was calculated as the average viscosity at the different shear rates.

### 2.7. Determination of the Strong Water-Binding Capacity

Strong water-binding capacity (SWBC) is defined as the amount of water bound by bran when an external force is applied, while bran and water are not in contact after application of the external force. A QIAprep Spin Miniprep Column (Qiagen, Hilden, Germany) was used, consisting of a filter as the upper part that is placed in an Eppendorf tube as the lower part. An amount of 50.0 mg of bran was weighed on the filter and 700 µL of water was added. Bran was hydrated for 60 min and afterwards centrifuged (10 min, 4000× *g*, room temperature). SWBC was than calculated as the amount of water that was bound by bran after the centrifugation step. To account for the water that was taken-up by the filter, three blank columns were included in the analysis and used for correction. The procedure was performed in triplicate. 

## 3. Results and Discussion

In this study, we investigated the scale-up of wheat bran extrusion-cooking and its effects on bran properties, by going from a BC21 extruder to a BC45 extruder and finally to a BC72 extruder. When upscaling, most general upscaling rules for extrusion-cooking were followed. The same equipment manufacturer (Clextral), design of extruder and high shear screw configuration were used [20]. Feed rate during extrusion-cooking was calculated based on the volume scale-up rule to avoid the extruder being force-fed or starve-fed [25]. The L/D ratio of the different extruders was kept as constant as possible (between 20 and 23.9), as changing the ratio would influence channel volume [20,25,27]. 

We did deviate from general upscaling rules for the process parameters in that we aimed to compare changes in properties of wheat bran processed using the same process parameters in different extruders instead of keeping product properties constant by varying process parameters. 

### 3.1. Impact of Extrusion Scale on Extruded Wheat Bran Physicochemical Properties: A Systematic Comparison of BC21 and BC45

The measured system parameters and physicochemical data of extruded wheat bran samples produced on the BC21 and BC45 extruders are summarised in Table 2. 

For all samples that were produced, extrusion-cooking with the BC45 always resulted in a higher pressure, a higher SME and a higher product temperature compared to extrusion-cooking on the BC21 (Table 2). The maximal SME was up to 55% higher during extrusion-cooking with the BC45 compared to the BC21. The highest pressure reached in the BC45 was 26% higher than in the BC21, while product temperature was 14% higher. The higher product temperatures in the industrial extruder can be related to the higher SME reached and thus higher shear, resulting in more heat generation due to friction. 

Differences in product temperature achieved with the various extrusion-cooking types (single-pass, double-pass, acid) were, however, less pronounced in the industrial-scale extruder as compared to the pilot-scale extruder. The trends are nonetheless the same: VHS23 and VHSD27 reached the highest product temperature in both extruders. Double-pass extrusion-cooking (VHSD27) resulted in the highest SME in the BC21 and the BC45, as the sample underwent the high shear treatment two times. The SME and pressure reached during acid extrusion-cooking were the lowest in both extruders, compared to the other samples. This can be linked to acid hydrolysis of the wheat bran structure, leading to less friction between the bran particles themselves and between bran and the extruder barrel, as explained by Roye, Chanvrier et al. [21]. This, in turn, led to less pressure build-up. 

Extruding on industrial scale resulted in wheat bran samples with a higher strong water-binding capacity (SWBC) (Table 2) than when using the pilot-scale extruder except for double-pass extrusion-cooking samples. Excluding the latter, SWBC reached values between 0.82 and 1.18 g/g dm for BC21 samples and values between 1.18 and 1.28 g/g dm were reached for BC45 samples, in comparison to the control wheat bran sample (0.88 g/g dm). For double-pass extrusion-cooking, SWBC values were 1.37 and 1.29 g/g dm for the BC21 and BC45, respectively. The second extrusion-pass increased the SWBC to a lesser extent using the BC45 compared to the BC21. It can be envisaged that after a maximum is reached, further degradation of the bran will lead to a decrease in SWBC. Varying the conditions of extrusion-cooking generated fewer differences in the SWBC of samples produced on an industrial scale as compared to the pilot scale. Trends were nevertheless still comparable. Lowering the total moisture content from 27% to 23% during single-pass extrusion-cooking decreased the SWBC in both extruders. Acid extrusion-cooking resulted in the lowest SWBC in both extruders, possibly due to the lower pressures reached, acid hydrolysis of the wheat bran structure or less cohesion of the suspension, as hypothesised by Roye, Chanvrier et al. [21]. It can be envisaged that a 46% (VHSD27 on the BC45) and 55% (VHSD27 in the BC21) increase in the SWBC compared to control wheat bran (0.88 g/g dm) will have techno–functional and physiological implications, as it will influence product quality [7] and faecal bulking [1]. 

For extractability and extract viscosity (Table 2), extruding on an industrial scale with the same process parameters as on a pilot scale resulted in higher extractabilities and extract viscosities. However, differences were rather low. The increase in extractability and extract viscosity during upscaling can be ascribed to the higher SME’s reached. Higher SME’s result in more solubilisation of large water-extractable arabinoxylan fragments, as shown during BC21 extrusion-cooking [14]. They can explain the observed trends during upscaling, as higher SME’s were reached during industrial-scale extrusion-cooking. Overall, higher SME’s can be expected to lead to a more pronounced macro- and microscopic structure degradation, resulting in the release of aleurone content, and solubilisation of starch and dietary fibre. When comparing the different extrusion types, extractability was the highest for VHSA2% in both extruders, while extract viscosity was the highest for VHSD27 in both extruders. Acid hydrolysis of the wheat bran structure explains the high extractability of VHSA2%, while further acid hydrolysis of the solubilised fragments explains why extract viscosity did not increase in proportion with extractability [21]. Despite the lower extractability of VHSD27 compared to VHSA2%, the extract viscosity was the highest for VHSD27 from both extruders (3.54 mPa·s in the BC21 and 4.75 mPa·s in the BC45). This was ascribed to the two passes of this sample through the extruder, solubilising long WE-AX chains. They drive the viscosity build-up [21]. 

Extrusion-cooking experiments were conducted once as the study is an exploratory study. However, the variability of the extrusion-cooking process was assessed by producing two samples in duplicate and comparing system parameters and wheat bran characteristics. Variability was found to be very small. The extrusion-cooking procedures as performed can, therefore, be expected to be reproducible and robust.

### 3.2. Comparing the Maximal Potential of Pilot- (BC21) and Industrial-Scale (BC45) Extrusion-Cooking to Modify Wheat Bran

To compare the maximal potential of the BC21 and BC45 extruders to modify wheat bran, a two-step approach was used. In the first instance, the lowest moisture content and the highest barrel set temperature that could be reached during extrusion-cooking were determined for both extruders. These two samples represent the most extreme samples that can be produced with each particular extruder. Decreasing the moisture content was chosen as an approach as it increases the SME and thus shear [14]. Increasing the barrel set temperature was chosen as another approach as it increases the product temperature. In a second step, wheat bran characteristics of samples produced during pilot-scale and industrial-scale extrusion-cooking were compared. The results give an indication of the potential of the extruders to modify wheat bran, aiming to increase structure degradation and loosen the wheat bran structure. 

With the BC21, the lowest reachable moisture content while assuring a stable production process was 23% when using the low-temperature profile. On the BC45 extruder, this was 19% (VHS19). Lowering the moisture content further led to blockage of the extruder or steam formation within the extruder, resulting in pressure and temperature fluctuations. The highest set temperature in the last barrel of the BC21 that still assured a stable production process was 145 °C, leading to a product temperature of a maximum of 130 °C. In the BC45, using 145 °C as the last barrel set temperature was not possible as such. Therefore, we chose to implement a preconditioning step, bringing the bran to 20% moisture. It was hypothesised that less free water would reduce the chance of steam formation in the extruder barrel at high temperatures. Moisture content was then further increased to 27% during extrusion-cooking itself. With this preconditioning step, a stable process was achieved at a barrel set temperature of 145 °C. Similar to pilot-scale extrusion-cooking, this also proved to be the highest reachable set temperature in the barrels. The product temperature reached for this sample (VHS27T, 156 °C) was nevertheless higher compared to pilot-scale extrusion-cooking. This can again be linked to the higher SME, leading to more heat generation by friction. The authors of [25] also concluded that temperatures were higher in a large-scale extruder due to the higher shear and the consequently higher viscous dissipation. However, the aim in that study was to produce similar products on different extruders, so the temperature profile in the barrels was lowered during large-scale production, which is possible as the cooling capacities are better on a bigger machine [28].

While extrusion-cooking on industrial scale always resulted in higher extract viscosities compared to pilot scale extrusion-cooking when the same process parameters were used (see Table 2), extract viscosity could be increased even more during industrial-scale extrusion-cooking because a lower moisture content could be sustained (19% in the BC45 versus 23% in the BC21). The extract viscosity for VHS19 was 6.69 mPa·s and the extractability was 22.6% dm. Decreasing the moisture content from 23% to 19% on an industrial scale thus increased the extract viscosity with 78% and increased the extractability with 23%. Decreasing the moisture content to 19% was not possible on the BC21. In comparison to control wheat bran, extrusion-cooking on an industrial scale using 19% of moisture increased extract viscosity by a factor of 5.2 and increased extractability by 34%. The high extract viscosity and extractability for VHS19 can be related to the high SME values that were reached (356 kJ/kg) and reflects the degradation of the wheat bran structure and solubilisation of viscosity-building poly- and oligomers. The SWBC of VHS19 (1.07 g/g dm) (BC45) was higher than control wheat bran (0.88 g/g dm), probably due to the high pressure that was reached (96 Bar) leading to water evaporation and bran expansion at the die. Lower viscoelasticity of the suspension of agglomerated bran particles due to intensive structure degradation can also contribute to the more pronounced expansion, as it lowers resistance against expansion [22]. 

Extruding under high barrel temperatures (VHS27T) on the BC21 led to wheat bran with an SWBC of 1.04 g/g dm (Table S1) while it was 1.45 g/g dm on the BC45 (Table 3). The SWBC thus increased by 39% due to upscaling and increased by 65% compared to control wheat bran. This effect can be linked to the higher product temperatures reached in the BC45 extruder, increasing the SWBC of wheat bran [14] and improving product expansion [22]. A high barrel temperature was shown to result in lower SME values [14], which can explain the low extractability of the VHS27T sample. 

These results show that the two extruders have a different maximal potentials to modify wheat bran characteristics and do so in a different way when operating at their limits for proper operation with regard to moisture content and last barrel set temperature, given the material used. The working range of the BC45 industrial-scale extruder was shown to be broader as lower moisture contents and higher product temperatures could be reached. Therefore, wheat bran can be modified more using an industrial-scale extruder compared to a pilot-scale extruder. However, when the aim is to produce products with comparable characteristics in a pilot- and industrial-scale extruder, the range of operating conditions is much more limited in an industrial-scale extruder [25]. 

The approach used in this article is different from general upscaling articles where product optimisation is mostly done on a pilot scale and upscaling is then performed, aiming to keep product properties constant by varying process parameters [20,27,29,30]. Djuric, Van Melkebeke et al. [31] compared two twin-screw extruders from different producers but with similar screw configurations on the wet granulation process and concluded that granule properties were dependent on the extruder type. However, when upscaling rules are followed, and process parameters are optimised, it is possible to obtain products with comparable characteristics [20,25,27]. Moreover, it is known that product transformations during extrusion-cooking correlate well with SME [14,30]. To obtain a comparable product on different extruders, one of the possible tactics is to keep SME constant during upscaling. However, if the aim is to induce as much shear as possible, it is more relevant to seek optimal process parameters on every extruder to obtain maximal SME. This was the approach followed in this study. 

### 3.3. Extrusion-Cooking with the BC72 Industrial-Scale Extruder: A Case Study

As a case study to evaluate the conclusions on upscaling from a pilot-scale extruder (BC21) to an industrial-scale extruder (BC45), an even larger extruder (BC72) was used for wheat bran extrusion-cooking. A sample was produced at the lowest moisture content reachable, i.e., 21.5%. Adaptation of the temperature profiles in the barrels to assure stability of the production process was required. This suggests that large-scale extrusion-cooking on the BC72 allows less variation in operating conditions and results more rapidly in instabilities. Upon using the adapted setting, a very high SME value, 522 kJ/kg, was reached in the BC72 compared to the BC21 and BC45. Pressure (65 Bar) and product temperature (149 °C) were nevertheless comparable with the BC45 samples, but higher than the BC21 samples. This shows that industrial-scale extrusion-cooking leads to higher SME’s, product temperatures and pressures compared to pilot-scale extrusion-cooking. The BC72 extruder increased the extractability of wheat bran (20.4%) compared to the BC21 and BC45 single-pass extruded samples, except VHS19, while the SWBC was comparable (1.21 g/g dm). Extractability was increased by 38% compared to the control sample. Extract viscosity (2.55 mPa·s), on the other hand, was higher compared to the control and the BC21 samples but lower compared to the BC45 samples. These effects could be ascribed to the high SME, leading to high shear. Structure degradation probably went hand in hand with depolymerisation of soluble compounds, resulting in a moderate increase in extract viscosity of the bran extruded using the BC72. 

Putting everything together, we can state that industrial-scale compared to pilot-scale extrusion-cooking led to more structure degradation (measured as extractability and extract viscosity) due to the higher SME’s reached. A comparable SWBC was obtained. As extrusion-cooking on a pilot and an industrial scale did not result in the same extent of wheat bran modification, literature results about the extent of bran modification should not be compared one-on-one. Physicochemical properties did evolve in the same direction if the type of extrusion-cooking and extrusion parameters were changed, irrespective of the scale of the extruder. Decreasing the moisture content increased SME and thus extractability and extract viscosity. Increasing barrel temperature increased product temperature and thereby SWBC. This shows that trends can be compared. The differentiating capacity that was achieved by changing the type of extrusion-cooking during pilot-scale extrusion-cooking was less pronounced during industrial-scale extrusion-cooking. However, lower moisture contents and a higher product temperature can be reached on an industrial-scale. In this way, structure degradation—measured as extractability and extract viscosity—and flash-off at the die—measured as SWBC—can be increased to a large extent on an industrial scale, beyond what is possible on a pilot scale. The results in this article show that conventional upscaling techniques, in which a product is developed on a pilot scale and then upscaled to an industrial extruder, aiming to keep product properties constant, do not allow the exploration of the full potential of an extruder to modify wheat bran. The extruder scale mainly determines the SME that can be reached and determines the potential to modify wheat bran.

## 4. Conclusions

Extrusion-cooking has considerable industrial potential as it can be executed on a large scale, is a cost-effective continuous short-time process and leads to the stabilisation of the final product. The results in this article show the potential of extrusion-cooking as a modification technique for wheat bran. Wheat bran was modified the most using industrial-scale extrusion-cooking. While the differentiating capacity at this scale was smaller compared to pilot-scale extrusion, it could be improved by decreasing the moisture content and increasing product temperature beyond what was possible in the pilot-scale extruder. When the aim is to degrade wheat bran as much as possible, the best strategy is to increase SME as much as possible by using an industrial-scale extruder at low moisture contents. Here, this approach resulted in the highest extractabilities and thus the most noticeable structure degradation on macro- and microscale. When the aim is to increase the interaction of bran with water, the best strategy is to increase product temperature as much as possible, using an industrial-scale extruder at high barrel temperatures. Care has to be taken not to overmodify the bran. The changes in wheat bran characteristics we observed in this study could possibly increase faecal bulk and normalise transit time, increase extract viscosity in the gastrointestinal tract and increase dietary fibre fermentability and thus SCFA production. Clinical studies are, however, needed to confirm these hypotheses. In addition, techno–functional characteristics and sensory attributes of the samples produced on an industrial scale can be assumed to differ from the samples produced on pilot-scale. These characteristics should thus be determined, evaluated and compared, but this was outside the scope of this article.

## Figures and Tables

**Table 1:** Overview of the samples produced on the BC21, BC45 and BC72 Clextral extruders. In the sample coding, VHS = very high shear, T = high temperature, D = double-pass extrusion, A2% = extrusion-cooking in the presence of 2% citric acid.

Extruder	Sample Coding	Moisture Content (%)	Last Barrel Set T (°C)	Acid Concentration (%)
**BC21**	VHS23	23	120	0
	VHS27	23	120	0
	VHS27T	27	145	0
	VHSD27	27	120	0
	VHSA2%	27	120	2
**BC45**	VHS19	19	120	0
	VHS23	23	120	0
	VHS27	23	120	0
	VHS27T	27	145	0
	VHSD27	27	120	0
	VHSA2%	27	120	2
**BC72**	VHS21.5	21.5	90	0

**Table 2:** System parameters, as measured during extrusion-cooking, and physicochemical properties of wheat bran extrudates generated using the BC21 (pilot scale) and the BC45 (industrial scale) extruders, and physicochemical properties of the control wheat bran sample. Sample coding is explained in Table 1. For double-pass extrusion-cooking, system parameters during the first and second extrusion pass are given. The data represent a single extrusion-cooking experiment starting from one batch of bran.

	Control	Single-Pass Extrusion				Double-Pass Extrusion		Acid Extrusion	
		VHS23		VHS27		VHSD27		VHSA2%	
		BC21	BC45	BC21	BC45	BC21	BC45	BC21	BC45
**Specific mechanical energy (kJ/kg)**	/	164	255	139	224	132/153	255/187	133	194
**Pressure (Bar)**	/	53	72	45	59	37/57	72/62	39	49
**Product T (°C)**	120	121	136	114	134	130/120	136/137	110	131
**Strong water-binding capacity (g/g dm)**	0.88 (±0.01)	1.05 (±0.03)	1.18 (±0.04)	0.95 (±0.08)	1.28 (±0.02)	1.37 (±0.03)	1.29 (±0.01)	0.82 (±0.03)	1.20 (±0.01)
Extract viscosity (mPa*s)	1.27 (±0.01)	2.96 (±0.02)	3.78 (±0.06)	1.92 (±0.03)	2.96 (±0.08)	3.54 (±0.03)	4.75 (±0.08)	2.55 (±0.02)	4.06 (±0.06)
**Extractability (% dm)**	16.9 (±0.4)	17.6 (±0.4)	18.3 (±0.2)	15.4 (±0.1)	16.8 (±0.4)	18.1 (±0.2)	18.8 (±0.2)	20.8 (±0.5)	23.0 (±0.3)

**Table 3:** System parameters and wheat bran characteristics of samples extruded using the BC45 and BC72 industrial-scale extruders at the lowest moisture contents possible and the highest last barrel set temperature possible, and physicochemical properties of the control wheat bran sample. Sample coding is explained in Table 1. The data represent a single extrusion-cooking experiment starting from one batch of bran. Abbreviations: SME, specific mechanical energy; T, temperature; SWBC, strong water-binding capacity

	Control	VHS19	VHS27T	VHS21.5
Extruder	/	BC45	BC45	BC72
Preconditioning (20% water)	No	No	Yes	No
SME (kJ/kg)	/	356	191	522
Pressure (Bar)	/	96	40	65
Product T (°C)	120	127	156	149
SWBC (g/g dm)	0.88 (±0.01)	1.07 (±0.08)	1.45 (±0.03)	1.21 (±0.03)
Extract viscosity (mPa·s)	1.27 (±0.01)	6.69 (±0.09)	3.29 (±0.06)	2.55 (±0.04)
Extractability (% dm)	16.9 (±0.4)	22.6 (±0.3)	16.3 (±0.2)	20.4 (±0.5)

## Supplementary Materials

The following are available online at https://www.mdpi.com/2304-8158/10/2/472/s1, Table S1: Overview of the measured process parameters and wheat bran characteristics of the samples extruded on a pilot scale (BC21). Results are discussed in Roye, Chanvrier et al. [21]. Sample coding is explained in Table 1. The data represent a single extrusion-cooking experiment starting from one batch of bran.

## Abbreviations

dm: dry matter, rpm: rotations per minute, SWBC: strong water-binding capacity, SME: specific mechanical energy, SCFA: short-chain fatty acids
