# Supplementary material for: Changing Wheat Bran Structural Properties by Extrusion-Cooking on a Pilot and Industrial Scale: A Comparative Study
Source: Foods. 2021 Feb 21;10(2):472. doi: 10.3390/foods10020472 (PMC7924859; doi:10.3390/foods10020472)
Supplement: Supplementary file 1 [file foods-10-00472-s001.pdf]

foods
MDPI

5. Supplementary Material

Table S1. Overview of the measured process parameters and wheat bran characteristics of the samples extruded on a pilot scale (BC21). Results are discussed in Roye, Chanvrier, et al. (2020). Sample coding is explained in Table 1. The data represent a single extrusion-cooking experiment starting from one batch of bran.

	SME (kJ/kg)	Pressure (Bar)	Product T (°C)	SWBC (g/g dm)	Extract viscosity (mPa*s)	Extractability (% dm)
**VHS23**	164	53	121	1.05 (±0.03)	2.96 (±0.03)	17.6 (±0.4)
**VHS27**	139	45	114	0.95 (±0.08)	1.92 (±0.03)	15.4 (±0.1)
**VHS27T**	132	37	130	1.03 (±0.03)	2.46 (±0.02)	16.6 (±0.6)
**VHSD27**	153	57	120	1.37 (±0.07)	3.54 (±0.02)	18.1 (±0.2)
**VHSA2%**	133	39	110	0.82 (±0.03)	2.55 (±0.03)	20.8 (±0.5)
